# The mechanism study of lentiviral vector carrying methioninase enhances the sensitivity of drug-resistant gastric cancer cells to Cisplatin

**DOI:** 10.1038/s41416-018-0043-8

**Published:** 2018-03-26

**Authors:** Lin Xin, Wei-Feng Yang, Hou-Ting Zhang, Yi-Fan Li, Chuan Liu

**Affiliations:** Department of General Surgery, The Second Affiliated Hospital of Nanchang University, Nanchang, 330006 Jiangxi China

**Keywords:** Cancer stem cells, Biochemistry

## Abstract

**Background:**

To investigate the mechanism of lentiviral vector carrying methioninase enhances the sensitivity of drug-resistant gastric cancer cells to Cisplatin.

**Methods:**

Death receptors, anti-apoptotic protein, NF-κB, and TRAIL pathway-related factors were detected. The influence of LV-METase transfection on cell viability and pathway-related proteins were assessed by MTT method and western blot, respectively. Different treatments (NF-κB or caspase-3 inhibitor induction, TRAIL supplement, etc.) were performed in gastric cancer cells and the above parameters were analysed. Moreover, the connection between miR-21 and NF-κB or caspase-8 was determined by Chip and luciferase assay, respectively. LV-METase transfection drug-resistant gastric cancer cells were injected subcutaneously into mice.

**Results:**

The expression of free MET, miR-21-5p, MDR1, P-gp, and DR5 was significantly increased in drug-resistant gastric cancer cell lines. When cells were transfected with LV-METase, intracellular TRAIL signalling was activated while NF-κB pathway was inhibited. Besides, enhanced TRAIL signalling or repressed NF-κB pathway can promote the sensitivity of drug-resistant strains to Cisplatin, and the combination shows more sensitive to sensitisation. LV-METase promoted TRAIL expression by reducing NF-κB, thereby contributing to the downregulation of P-gp and enhancing the susceptibility of drug-resistant gastric cancer cells to Cisplatin. Furthermore, miR-21 regulated by NF-κB mediated the expression of P-gp protein via inhibiting caspase-8, thus regulating Cisplatin-induced cell death.

**Conclusions:**

Our results suggest that LV-METase has potential as a therapeutic agent for gastric cancer treatment.

## Introduction

Although the progress of medical technology has been made to improve gastric cancer outcomes, stomach cancer is still the fourth most common malignancies in the world. The five-year overall survival rate of stomach cancer patients is only about 35%, and it is the main cause of cancer-related deaths both in men and women for several decades. Moreover, one of the major reasons for deaths of gastric cancer is multidrug resistance,^1^ and it is a major obstacle to successful cancer chemotherapy, but the potential molecular mechanisms of multidrug resistance of gastric cancer is not completely clear and new targets with increased therapeutic efficacy to treat gastric cancer are of great demand.

Methioninase (METase) is a pyridoxal-l-phosphate (PLP)-dependent enzyme with four 43 kDa subunits, is utilised as a therapeutic option for various carcinomas. In nude mice, intraperitoneal injection of METase inhibits the growth of Yoshida sarcoma and slows the development of H460 human non-small cell lung cancer.^2^ Furthermore, METase also has good effects on the treatment of tumour-bearing mice, including tumours with multiple drug resistance.^3^ METase starvation therapy, such as methionine-free diets or methionine-depleted total parenteral nutrition treatment, prolonging the survival time of tumour-bearing rodents.^4^ It has been previously demonstrated that METase combined with chemotherapeutic agents such as Cisplatin, urea, and vincristine show synergistic antitumour effects in rodent and human tumour models.^5,6^ In addition, methionine-free total parenteral nutrition in combination with chemotherapeutic drugs also extend the survival of high-stage gastric cancer patients.^7^ METase from *Pseudomonas putida*, which degrades extracellular methionine to α-ketobutyrate, ammonia, and methanethiol, has been demonstrated to have antitumour efficacy in vitro and in vivo.^6,8^ Nevertheless, the clinical significance and biological mechanisms of METase in the progression of gastric cancer remain largely unknown.

Tumor necrosis factor-related apoptosis-inducing ligand (TRAIL) is a member of tumour necrosis factor (TNF) super family. It is considered to be a promising anticancer agent, and it can selectively induce cell death in transformed cells but no damage to normal cells.^9^ Moreover, TRAIL acts as an extracellular activator to initiates apoptotic signals by binding to cell surface death receptors (DRs), including DR4 (also known as TRAIL-R1) and DR5 (also known as TRAIL-R2), thus immediately leading to receptor aggregation and recruitment of Fas-associated death domain (FADD) followed by caspase-8 and caspase-3 activation.^10^ Drugs targeting TRAIL signalling, including recombinant TRAIL and agonistic antibodies, have been demonstrated with robust anticancer activity in a number of preclinical studies.^11–13^

Recently, more findings suggested that multiple cell survival signals, mainly including mitogen-activated protein kinase (MAPK) pathway, phosphatidylinositol 3-kinase/Akt (PI3K/AKT) transduction pathway, and nuclear factor-κB (NF-κB), play important role in regulation of TRAIL signalling.^14–16^ Among them, NF-κB acts as a well-known transcription factor, protects cells from apoptosis by the activation of survival factors such as anti-apoptotic proteins.^17^ It has been shown that inhibition of NF-κB in HeLa cells can sensitise the cancer cells to TNF-α- and TRAIL-induced apoptosis.^18–20^ Furthermore, it has been reported that NF-κB pathway is involved in melatonin-induced apoptosis in human gastric cancer SGC7901 cells.^21^ Liu et al. found that Fas-associated factor 1 inhibits tumour growth by suppressing Helicobacter pylori-induced activation of NF-κB signalling pathway in human gastric carcinoma.^22^ However, whether NF-κB is associated with the antitumour efficacy of METase remains unclear.

Therefore, the present report demonstrated the efficacy of METase on Cisplatin-resistant and Cisplatin-induced cell death. Furthermore, we also investigated the mechanism of METase enhances the sensitivity of drug-resistant gastric cancer cells to Cisplatin.

## Materials and methods

### Cell line and culture

The human gastric cancer cell lines (BGC823 and SGC7901) were purchased from the Type Culture Collection of the Chinese Academy of Sciences (Shanghai, China). All the cells were cultured in RPMI 1640 medium supplemented with 10% of foetal bovine serum, 100 U/ml of penicillin and 100 mg/ml of streptomycin. The cells were grown at 37 °C in a humidified incubator with 5% CO_2_. Cisplatin was obtained from Sigma-Aldrich. The Cisplatin-resistant BGC823/DDP cells were developed from the parental BGC823 cells that were subjected to persistent gradient exposure to Cisplatin, through increasing Cisplatin concentration from 0.06 μg/ml until the cells acquired resistance to 1 μg/ml. The Cisplatin-resistant SGC7901/DDP cells were obtained by the same way. After that, Cisplatin half maximal inhibitory concentration (IC_50_) values were analysed.

### CCK-8 cell viability assay

Cell Counting Kit-8 (CCK-8) assay was performed to detect the cell viability according to the manufacturer’s instructions. Indicated cells were seeded into 96-well plate at 4 × 10^3^ cells per well with 100 μl cultured medium. CCK-8 solution (10 μl) was added to each well and the plate was incubated for 2 h at 37 °C. The absorbance was recorded at 450 nm on a SpectraMax M5 microplate reader (Molecular Devices, USA).

### RNA extration and qRT-PCR

Total RNA was isolated using the Trizol reagent (Vigorous Biotech, China) following the manufacturer’s instructions. An aliquot of 2 μg of total RNA was subjected to reverse transcription with M-MLV reverse transcriptase (Promega, Madison, WI). Quantitative real-time PCR (qRT-PCR) reactions were carried out on an Mx3000P real-time PCR system (Stratagene, USA). To correct for the experimental variations between samples, β-actin and U6 were used as the corresponding internal controls. All mRNA levels were calculated using the 2^−△△CT^ method. The results were analysed by Mx3000P real-time PCR software version 2.00. PCR primers were shown in Supplemental Table.

### Western blot analysis

For western blot, tissues and cells were rinsed in ice-cold phosphate buffer and lysed in RIPA lysis buffer to collect proteins. Samples were then centrifuged for 20 min at 12,000 × *g*. The concentration of protein extracts was determined by the Bradford method. An equal amount of protein was elecrophoresed on 12% SDS-PAGE and transferred to PVDF membranes. Membranes were blocked in non-fat dry 5% milk in Tris-HCl buffered saline and incubated with each primary antibody overnight at 4 °C. Next, all blots were incubated with horseradish peroxidase (HRP)-conjugated secondary antibody (1:3000 goat anti-rabbit, Abcam) for 1 h. Immunoblots were visualised by enhanced chemiluminescence (ECL kit, Santa Cruz Biotechnology) and recorded with ChemImager 5500 V2.03 software. The relative integrated density values were calculated with β-actin as an internal control.

### Flow cytometric analysis

Cell apoptosis was assessed using the Annexin V-FITC Apoptosis Detection Kit (JingMei Biotech, Beijing, China), according to the manufacturer’s instructions. In brief, 100 μl cell suspension containing 1 × 10^5^ cells were prepared. Then, 5 μl Annexin V-FITC and 10 μl propidium iodide (PI) (20 μg/ml) were added and incubated in the dark for 15 min and the ratio of apoptotic cells were analysed by FACS Calibur flow cytometry (Becton Dickinson, Franklin Lakes, NJ) equipped with the ModiFit LT v2.0 software.

### Cell transfection

The experiment of cell transfection was performed with Lipofectamine 2000 Transfection Reagent (Thermo Fisher Scientific) in accordance with the directions of reagent. Methioninase lentiviral vector (LV-METase) and negative control lentiviral vector (LV-NC) were purchased from Shanghai Cancer Institute, China. miR-21-5p mimic and mimic negative control, miR-21-5p inhibitor, and inhibitor negative control were purchased from RiboBio Co., Ltd. (Guangzhou, China). Cells were cultured in 96-well plates with a concentration of 2 × 10^4^ cells/well for 24 h and transfected with the appropriate expression vectors at ~70% confluence. The applicable stably transfected cell lines were established by selection with G418 screening.

### Chromatin immunoprecipitation (ChIP) assay

Chromatin immunoprecipitation (ChIP) was performed using a ChIP assay kit according to the manufacturer’s instructions. BGC823/DDP cells or SGC7901/DDP cells were fixed in formaldehyde, and sonicated to prepare the chromatin fragments. Antibodies against acetylated histone H3 and H4 (Millipore) were used to evaluate histone modifications associated with the miR-21 promoter. Enrichment of miR-21 promoter fragments was quantified by qRT-qPCR with the primers as follows: forward: 5′-ATTGGAGTGGATGGGTTCTGCC-3′ and reverse: 5′-AAGTATGTCAGTGCAAAGTATGG-3′.

### Luciferase assay

A luciferase reporter assay was used to verify that the complementary sequence of miR-21 bound to the 3′-UTR of caspase-8 mRNA. A fragment of caspase-8 mRNA, which included the binding site of miR-21, was cloned into the pMIR-report luciferase reporter vector. BGC823/DDP cells or SGC7901/DDP cells were seeded in a 24-well plate so that they were 50% confluent at the time of transfection. The luciferase reporter vector and miR-21 mimic were co-transfected in cells using calcium phosphate precipitation. Relative luciferase activity was measured by the Dual-Luciferase Reporter Assay (Promega, Madison, WI, USA) on a LuminoskanTM Ascent Microplate Luminometer (Thermo Fisher Scientific, Waltham, MA, USA).

### Nude mouse xenograft

Female BALB/c nude mice (*n* = 20) were purchased from the Animal Experimental Center of The Second Affiliated Hospital of Nanchang University, and the methods of animal experiment were carried out strictly in accordance with the guidelines of the experimental animal management of The Second Affiliated Hospital of Nanchang University. BGC823/DDP or SGC7901/DDP cells transfected with LV-METase or LV-NC were injected subcutaneously into nude mice. Following that, Cisplatin at the concentration of 5 mg/kg was intraperitoneal injected twice per week for 4 weeks. At the end of the procedure, tumour volume of all mice was measured and calculated by the formula: volume (mm^3^) = length × width^2^/2.

### Statistical analysis

Data are presented as the mean ± standard deviation (SD). All experimental results were statistically analysed with Student’s *t*-test or one-way analysis of variance (ANOVA). All statistical analyses were performed with SPSS 18.0 statistical software, with *P* < 0.05 considered as statistically significant.

## Results

### Expression of related factors in drug-resistant gastric cancer cells

**Fig. 1:**
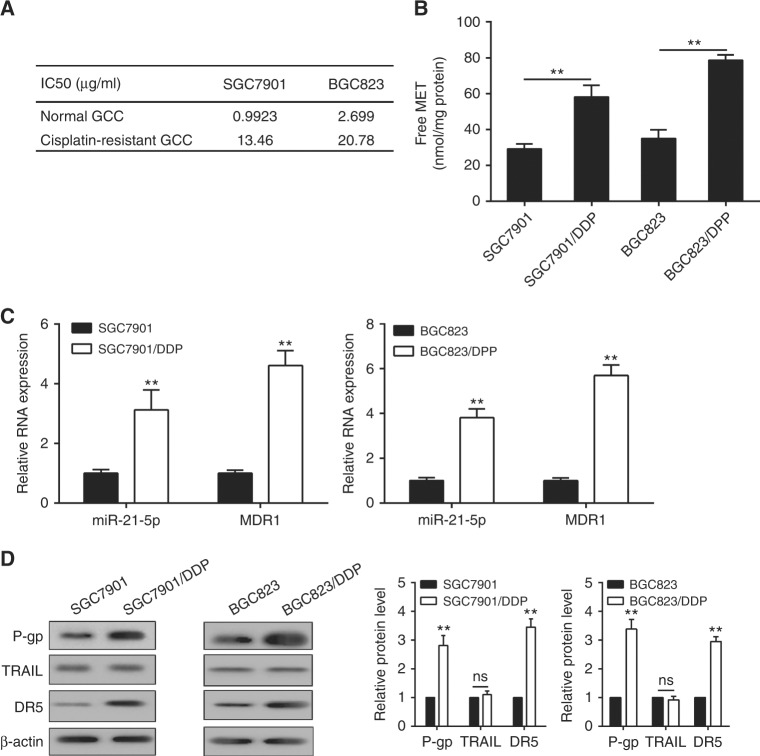
Expression of related factors in drug-resistant gastric cancer cells. **a** IC_50_ in normal cells and Cisplatin-resistant cells; **b** free METase in normal cells and Cisplatin-resistant cells; **c** relative RNA expression of miR-21-5p and MDR1 in normal cells and Cisplatin-resistant cells; **d** relevant factors protein level in cells. DDP means Cisplatin, β-actin, and U6 were used as the corresponding internal controls. Each sample was repeated three times. **P < 0.01

Cell viability was significantly increased in drug-resistant gastric cancer cell lines SGC7901/DDP (Supplementary Figure 1A) and BGC823/DDP (Supplementary Figure 1B), as compared with normal cells. With the treatment of different concentrations of Cisplatin, IC_50_ values were analysed and presented in Fig. 1a. Moreover, free MET concentration (Fig. 1b) and relative RNA expression of miR-21-5p, MDR1 genes (Fig. 1c) were obviously elevated in SGC7901/DDP and BGC823/DDP cells. The protein production of P-gp and DR5 was highly expressed in drug-resistant cells than those in the control group. There was no significant alternation in TRAIL protein expression, and no TRAIL was detected in the culture medium (data was not shown) (Fig. 1d). Additionally, the cell apoptosis of SGC7901, SGC7901/DDP, BGC823, and BGC823/DDP was increased in a concentration-dependent manner of Cisplatin (the concentrations were 0, 1, 8, and 16 μg/ml), while the cell apoptosis showed no significant difference between resistant cells and control (Cisplatin concentration = 0) when the Cisplatin concentration reached to 1 μg/ml (Supplementary Figure 1C).

### Effects of LV-METase transfection on intracellular TRAIL and NF-κB pathway in drug-resistant gastric cancer cells

**Fig. 2:**
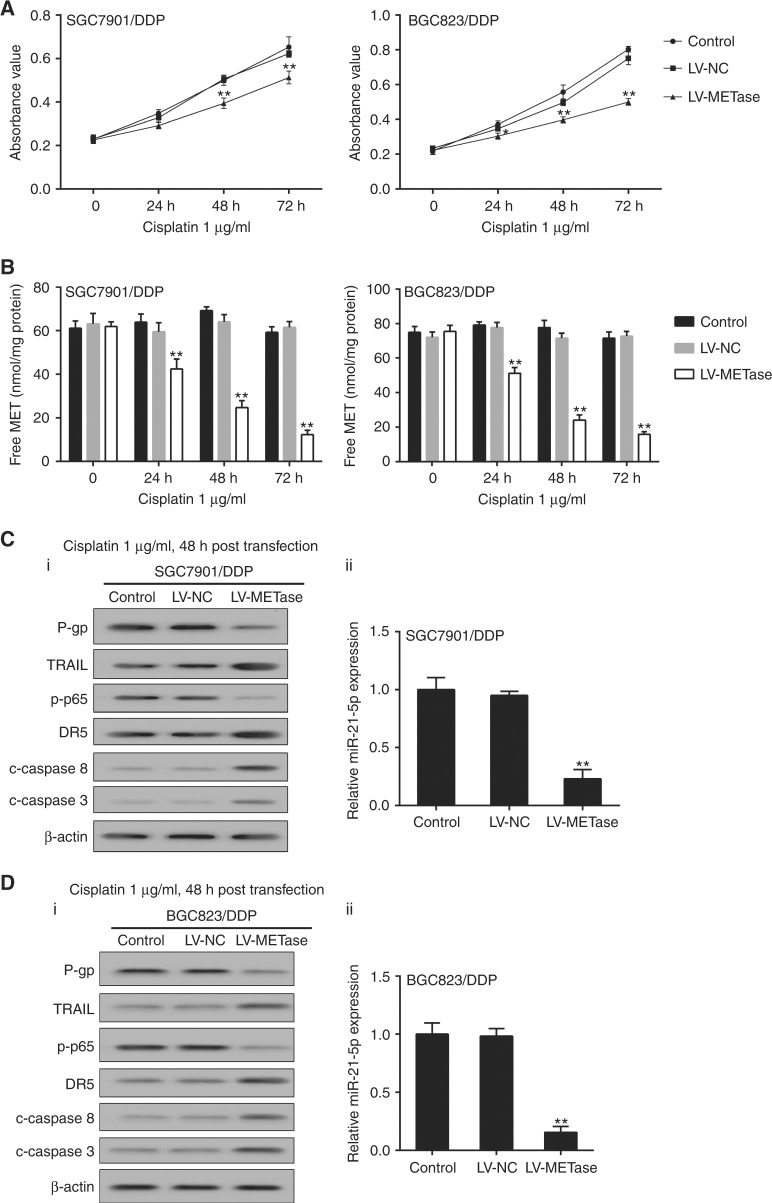
Effects of LV-METase transfection on intracellular TRAIL and NF-κB pathway in drug-resistant gastric cancer cells. Cells were transfected with LV-METase, then we detected **a** cell viability in SGC7901/DDP and BGC823 /DDP cells; **b** free METase in Cisplatin-resistant cells; **c**_i_ and **d**_i_ intracellular TRAIL and NF-κB pathway-related proteins expression in SGC7901/DDP and BGC823 /DDP cells; **c**_ii_, **d**_ii_ relative RNA expression of miR-21-5p in Cisplatin-resistant cells; DDP means Cisplatin, β-actin was acted as the internal control in western blot, and U6 was used as the reference. Each sample was repeated three times. **P < 0.01

When compared with control or LV-NC group, the cell growth ratio and free MET level in transfected gastric cancer cells transfected with LV-METase were significantly decreased at 24, 48, and 72 h and showing a time-dependent manner (Fig. 2a, b). In addition, western blot analysis at protein level presented that P-gp and p-p65 were obviously reduced, along with the decreased miR-21-5p detected by qRT-PCR; while cleaved-caspase-8/3, DR5, and cleaved-caspase-8/3 were drastically promoted in LV-METase transfection SGC7901/DDP (Fig. 2c) and BGC823/DDP (Fig. 2d). Besides, the level of membrane TRAIL assessed by flow cytometric was significantly increased in SGC7901/DDP and BGC823/DDP cells transfected with LV-METase (Supplementary Figure 2A).

### Effects of enhanced TRAIL or suppressed NF-κB pathway on Cisplatin sensitivity in drug-resistant gastric cancer cells

**Fig. 3:**
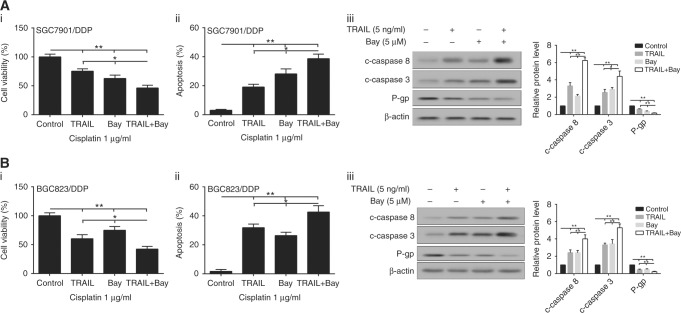
Effects of enhanced TRAIL or suppressed NF-κB pathway on Cisplatin sensitivity in drug-resistant gastric cancer cells. Cells were treated with TRAIL or Bay (NF-κB inhibitor), then we detected cell viability in SGC7901/DDP and BGC823/DDP cells (**a**_i_, **b**_i_); **a**_ii_, **b**_ii_ the percentage of apoptosis in Cisplatin-resistant cells; **a**_iii_, **b**_iii_ the expression of apoptosis proteins and P-gp in SGC7901/DDP and BGC823 /DDP cells. DDP means Cisplatin, β-actin was acted as the internal control in western blot. Each sample was repeated three times. **P < 0.01

TRAIL or Bay (a NF-κB inhibitor) treatment successfully curbed cell viability in drug-resistant gastric cancer cell lines, including SGC7901/DDP and BGC823/DDP (Fig. 3a_i_, b_i_), and the inhibitory effect was the most significant in TRAIL + Bay group. By contrast, TRAIL, Bay, or combined induction could significantly upregulate the number of apoptosis cells in SGC7901/DDP and BGC823/DDP (Fig. 3a_ii_, b_ii_). Further studies confirmed that TRAIL (5 ng/ml), Bay (5 μM), or combined treatment promoted the expression of cleaved-caspase-8/3, but inhibited the expression of P-gp in drug-resistant gastric cancer cells SGC7901/DDP and BGC823/DDP (Fig. 3a_iii_, b_iii_), as compared with normal cells.

### METase downregulated the level of P-gp protein by TRAIL pathway to promote cisplatin sensitivity in drug-resistant gastric cancer cells

**Fig. 4:**
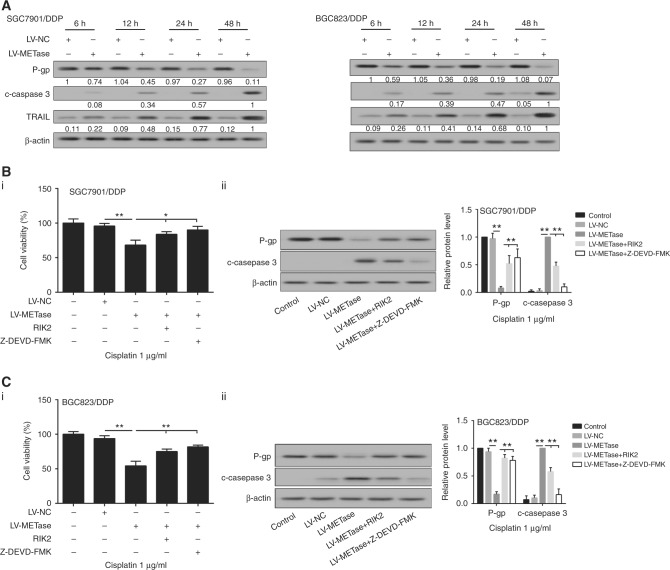
METase downregulated the level of P-gp protein by TRAIL pathway to promote Cisplatin sensitivity in drug-resistant gastric cancer cells. Cells were transfected with LV-METase and exposure to 1 µg/ml Cisplatin, then we detected **a** protein expression of TRAIL, P-gp, and c-caspase-3 in Cisplatin-resistant cells; cells were treated with Z-DEVD-FMK (caspase-3 inhibitor), then we assessed **b** the expression of P-gp in SGC7901/DDP and BGC823 /DDP cells; cells were also treated with Z-DEVD-FMK (caspase-3 inhibitor) or RIK2 (TRAIL inhibitor), then we determined **c**_i_, **d**_i_ cell viability in Cisplatin-resistant cells; **c**_ii_, **d**_ii_ the protein level of P-gp and c-caspase-3 in cells. DDP means Cisplatin, β-actin was acted as the internal control in western blot. Each sample was repeated three times. **P < 0.01

Drug-resistant gastric cancer cell lines SGC7901/DDP and BGC823/DDP were transfected with LV-METase and exposed to Cisplatin at the dose of 1 μg/ml. The protein level of P-gp, cleaved-caspase-3, and TRAIL was detected at 6, 12, 24, and 48 h. Western blot analysis indicated that LV-METase transfection led to the acceleration of cleaved-caspase-3 and TRAIL, accompanied by decreased P-gp expression (Fig. 4a). Moreover, SGC7901/DDP and BGC823/DDP cell lines were pre-treated with different dosages of TRAIL (1, 5, and 10 ng/ml), and P-gp protein level was obviously downregulated in a concentration-dependent manner. Then, 50 μM Z-DEVD-FMK (cleaved-caspase-3 inhibitor) was supplemented into cells and the reduced expression of P-gp induced by TRAIL could be strongly eliminated by Z-DEVD-FMK treatment (Fig. 4b). Meanwhile, RIK2 (TRAIL inhibitor) was inserted at the same time of LV-METase transfection and results showed that decreased cell viability and P-gp protein level was obviously reversed by RIK2 or Z-DEVD-FMK induction, while the expression of cleaved-caspase-3 showed the opposite outcome in SGC7901/DDP and BGC823/DDP cells (Fig. 4c). Additionally, the expression of P-gp was decreased with the increase of TRAIL, while Z-DEVD-FMK supplementation reversed the expression of P-gp, indicating TRAIL-deteriorated P-gp through regulating caspase-3 (Supplementary Figure 2B). Furthermore, to block the TRAIL signalling by RIK2 or TRAIL blocker or block the caspase-3 by using Z-DEVD-FMK could reverse the effects of METase on cell apoptosis (Supplementary Figure 1D).

### METase upregulated the expression of DR5 protein by NF-κB pathway to promote P-gp degradation in drug-resistant gastric cancer cells

**Fig. 5:**
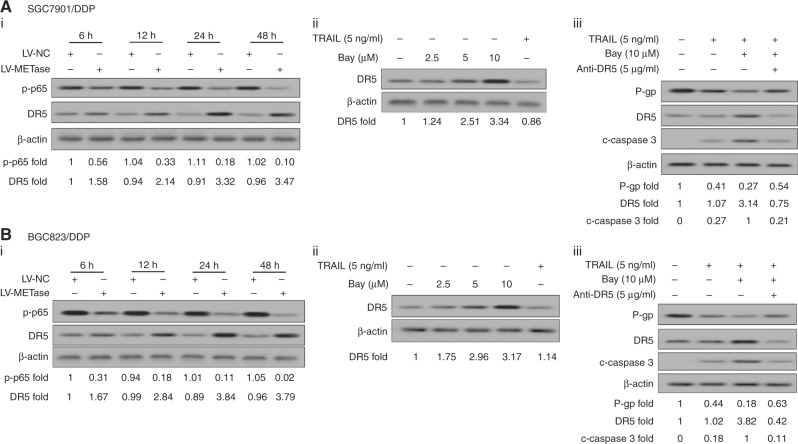
METase upregulated the expression of DR5 protein by NF-κB pathway to promote P-gp degradation in drug-resistant gastric cancer cells. Cells were transfected with LV-METase. **a**_i_, **b**_i_ The protein level of p-p65 and DR5 was assessed at 6, 12, 24, and 48 h in SGC7901/DDP and BGC823 /DDP cells; cells were treated with Bay or TRAIL. **a**_ii_, **b**_ii_ The protein level of DR5 was detected in Cisplatin-resistant cells; cells were treated with Bay or TRAIL or anti-DR5. **a**_iii_, **b**_iii_ The protein level of P-gp, c-caspase-3, and DR5 was detected in Cisplatin-resistant cells. DDP means Cisplatin, β-actin was acted as the internal control in western blot. Each sample was repeated three times. **P < 0.01

LV-METase transfection was performed in drug-resistant gastric cancer cell lines SGC7901/DDP and BGC823/DDP, at the same time with 1 μg/ml Cisplatin exposure. The protein level of DR5 and p-p65 was detected by western blot analysis, and results presented that the expression of DR5 protein was promoted, but the level of p-p65 was simultaneously inhibited at 6, 12, 24, and 48 h in a time-dependent manner (Fig. 5a_i_, b_i_). In addition, DR5 protein level was highly expressed in the presence of Bay (2.5, 5, and 10 μM); however, its expression was strongly decreased when cells induced by TRAIL (5 ng/ml) without Bay treatment (Fig. 5a_ii_, b_ii_). Then, cells were co-administrated by TRAIL (5 ng/ml), Bay (10 μM), and anti-DR5 (5 μg/ml). As shown in Fig. 5a_iii_, 5b_iii_, the level of P-gp was inhibited by TRAIL treatment, and this inhibition was more apparent in TRAIL + Bay group, while this effect could be eliminated in TRAIL + Bay + anti-DR5 group. However, the expression of DR5 and cleaved-caspase-3 productions presented the opposite trend in SGC7901/DDP and BGC823/DDP cells.

### METase regulated Cisplatin sensitivity by NF-κB/miR-21 pathway in drug-resistant gastric cancer cells

**Fig. 6:**
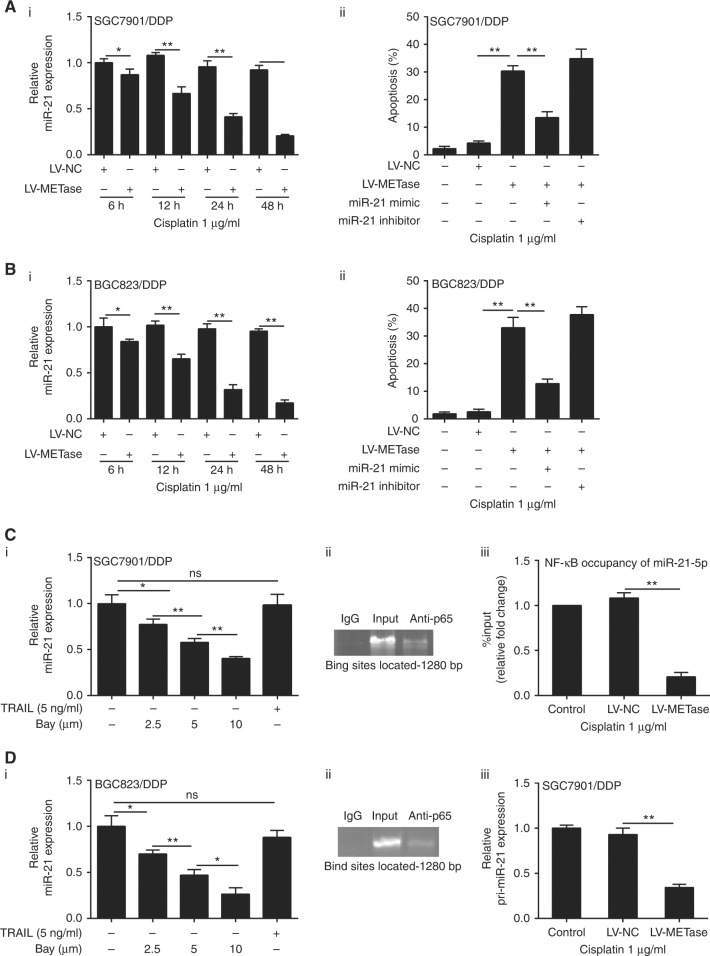
METase regulated cisplatin sensitivity by NF-κB/miR-21 pathway in drug-resistant gastric cancer cells. Cells were transfected with LV-METase or miR-21 mimic, then we detected **a**_i_, **b**_i_ relative miR-21 expression in Cisplatin-resistant cells. **a**_ii_ and **b**_ii_ The percentage of apoptosis in cells; cells were treated with Bay or TRAIL, then we assessed **c**_i_ and **d**_i_ relative miR-21 expression in SGC7901/DDP and BGC823 /DDP cells; **c**_ii_, **d**_ii_ Combination of p65 and miR-21 promoters through Chip method; cells were transfected with LV-METase and exposed to 1 µg/ml Cisplatin, then we detected **c**_iii_, **d**_iii_ percentage of NF-κB occupancy of miR-21-5p in Cisplatin-resistant cells; **c**_iv_, **d**_iv_ Relative miR-21 expression in cells. DDP means Cisplatin, and U6 was used as the reference. Each sample was repeated three times. *P < 0.05, **P < 0.01. miRNC is the abbreviation of miRNA control

The relative miR-21 expression and apoptosis cell number were detected using qRT-PCR and flow cytometric method, respectively. In this study, we found that when compared with control group, the mRNA level of miR-21 in LV-METase-transfected group was significantly downregulated at 6, 12, 24, and 48 h and showing a time–effect relation (Fig 6a_i_, b_i_). Moreover, the number of apoptosis cells was obviously upregulated in cells transfected with LV-METase, as compared with LV-NC group; while the percentage of apoptosis cells was markedly reduced in drug-resistant gastric cancer cells transfected with miR-21 mimic than that in the LV-METase group. However, the cell apoptosis between the treatments of METase + miR-21 inhibitor and METase showed no significant difference, which might due to that the expression of miR-21 was less, while miR-21 inhibitor showed no significant effect on cells (Fig 6a_ii_, b_ii_). In addition, cells were induced by Bay or TRAIL (5 ng/ml) and results showed that miR-21 was obviously decreased in a dose-dependent pattern in the presence of Bay, and TRAIL alone treatment did not influence relative miR-21 expression (Figure 6c_i_, d_i_). Through ChIP method, we found that p65 can be combined with miR-21 promoter (Figure 6c_ii_, d_ii_). The percentage of NF-κB occupancy of miR-21-5p and relative pri-miR-21 expression was evidently decreased in cells transfected with LV-METase, as compared with LV-NC group (Figure 6c_iii, iv_, d_iii, iv_). Additionally, the combination of METase and Cisplatin dramatically suppressed cell viability (Supplementary Figure 3A), promoted cell apoptosis (Supplementary Figure 3B), and decreased the expression of p-p65 (Supplementary Figure 3C).

### miR-21 inhibited caspase-8 level and increased P-gp expression thus regulating cisplatin sensitivity in drug-resistant gastric cancer cells

**Fig. 7:**
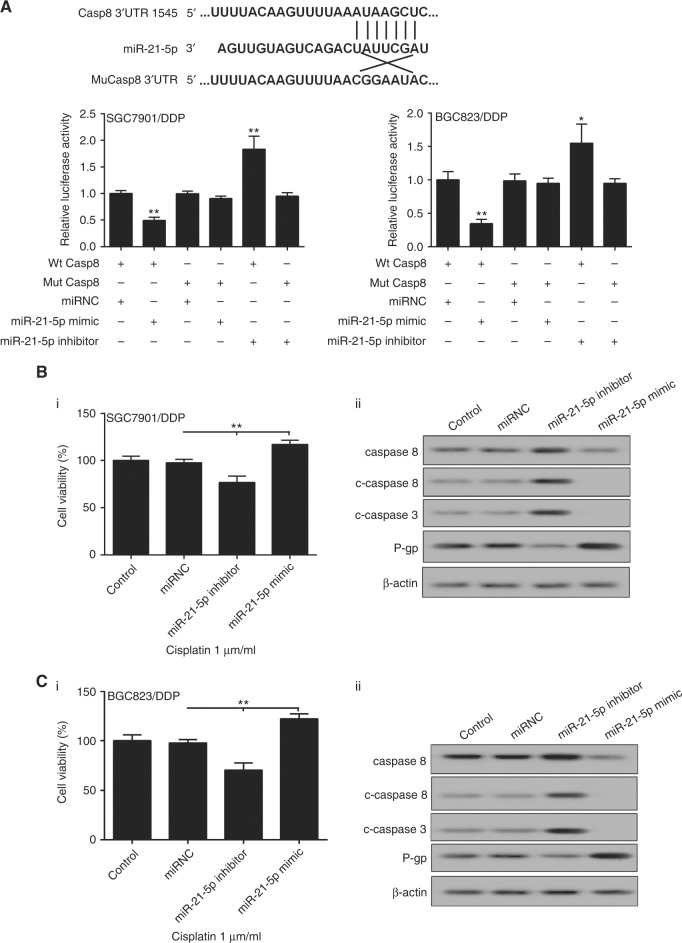
miR-21 inhibited caspase-8 level and increased P-gp expression thus regulating Cisplatin sensitivity in drug-resistant gastric cancer cells. **a** Luciferase experiments verified that miR-21 regulates caspase-8; cells were transfected with miR-21-5p inhibitor or miR-21-5p mimic, then we determined **b**_i_, **c**_i_ cell viability in SGC7901/DDP and BGC823 /DDP cells; **b**_ii_, **c**_ii_ the protein expression of P-gp, c-caspase-8, and c-caspase-3 in Cisplatin-resistant cells. DDP means Cisplatin, β-actin was acted as the internal control in western blot. Each sample was repeated three times. **P < 0.01

Biological prediction analysis indicated the complementarily of miR-21-5p with the 3′-UTR of caspase-8. Additionally, SGC7901/DDP and BGC823/DDP cells were transfected with miR-21-5p mimic and the luciferase activity of a pMIR-Report construct containing the caspase-8 3′-UTR was significantly repressed in the cells overexpressing miR-21-5p (Fig. 7a). Next, we further investigated whether miR-21-5p could influence the proliferation of drug-resistant gastric cancer cells. The cell viability of SGC7901/DDP and BGC823/DDP cells transfected with miR-21-5p inhibitor was significantly lower than that of the control cells, while miR-21-5p overexpression upregulated cell viability (Fig. 7b_i_, c_i_). Further studies indicated that miR-21-5p silencing promoted the protein expression of caspase-8, cleaved-caspase-8/3, but inhibited the expression of P-gp. By contrast, the expression of related factors in cells transfected with miR-21-5p mimic was just opposite (Fig. 7b_ii_, c_ii_). Additionally, miR-21 knockdown significantly promoted cell apoptosis, while miR-21 overexpression decreased cell apoptosis in SGC7901/DDP cells, while the expression of miR-21 did not affect cell apoptosis of BGC823/DDP cells (Supplementary Figure 1E).

### LV-METase enhanced Cisplatin sensitivity and inhibited tumour growth in mice

**Fig. 8:**
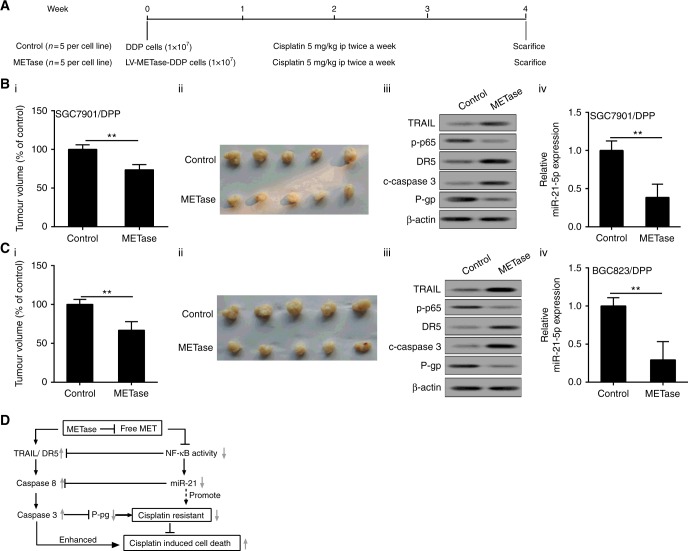
LV-METase enhanced cisplatin sensitivity and inhibited tumour growth in mice. **a** The procedure sketch of animal experiment in vivo; METase was transfected into mice, then we assessed **b**_i_, **c**_i_ tumour volume; **b**_ii_, **c**_ii_ representative pictures of tumour; **b**_iii_, **c**_iii_ the protein level of TRAIL, DR5, c-caspase-3, P-gp, and p- p65; **b**_iv_, **c**_iv_ relative miR-21 expression; **d** the whole experimental design diagram. β-actin and U6 were acted as the internal control. **P < 0.01

The experimental design of the current study was presented in Fig. 8a. At the end of the procedure, tumour volume of all mice was measured and calculated. Results showed that LV-METase transfection could significantly inhibit the growth of grafted tumour in vivo (Figure 8b_i, ii_, c_i, ii_). The expression of P-gp, p-p65, and miR-21 in tissues was obviously decreased, while the protein level of TRAIL, DR5, and c-caspase-3 was remarkably increased in METase transfection cells than those of controls (Fig. 8b_iii, iv_, c_iii, iv_). Finally, schematic overview of the present research was also provided in Fig. 8d.

## Discussion

Despite considerable progress has been made in the field of cisplatin resistance, the underlying mechanisms are not clear. In this study, we found that LV-METase enhances cell death in Cisplatin-stimulated conditions by a mechanism involving the inhibition of NF-κB/miR-21 pathway and the activation of TRAIL pathway (Fig. 8d). These effects were associated with LV-METase-mediated cell apoptosis in gastric cancer resistance cell, which may be derived from a combination effect on the reduction apoptosis genes. Our results show that LV-METase could increase Cisplatin-induced cell death via NF-κB and TRAIL pathways.

TRAIL, a member of TNF super family, can induce apoptosis of tumour cells with high selectivity, but shows no obvious damage toward normal cells,^23^ which has a promising future in the treatment of various cancers. In addition, TRAIL also presents strong apoptotic effects on multidrug resistance cells, which can increase the sensitivity of drug resistance cells to related chemotherapeutic agents.^24^ It has also been demonstrated that decreasing the expression of multidrug resistance-related protein of P-glycoprotein (P-gp) and multidrug resistance protein-1 (MRP1) can increase the sensitivity of oesophageal squamous cell carcinoma (ESCC) cell line Ec9706 to chemotherapeutic drugs.^25^ Seo et al. found that TRAIL can increase the cytotoxicity of chemotherapeutic drugs against human leukemia cell line CEM/VBL by downregulating the expression of P-gp.^24^ In the current study, TRAIL could bind to DR5 and further initiate apoptotic signals, thus resulting in upregulated caspase-8 and caspase-3 expression and downregulated P-gp expression, finally enhancing Cisplatin-induced cell death and alleviating Cisplatin resistant. Moreover, when drug-resistant gastric cancer cells SGC7901/DDP and BGC823/DDP were transfected with LV-METase, intracellular TRAIL signalling pathway was activated and LV-METase downregulated the expression of P-gp protein to promote Cisplatin sensitivity in drug-resistant gastric cancer cells.

Mounting documents have indicated that NF-κB participates in the regulation of tumour cell growth and metastasis,^26^ however, whether it is involved in the progression of chemotherapy agents induced apoptosis in drug-resistant gastric cancer cells remains rarely reported. In our research, the expression of p-p65 was downregulated and NF-κB pathway was inhibited, and cell death was obviously exacerbated, thus leading to Cisplatin resistant. Our findings were similar with the opinions of other scholars, namely NF-κB plays a negative regulatory role in the process of TRAIL apoptotic signalling.^27^ Furthermore, after drug-resistant gastric cancer cells were transfected with LV-METase, NF-κB pathway was inactivated and LV-METase inhibited NF-κB activity, ultimately alleviating Cisplatin-resistant and promoting Cisplatin-induced cell death.

Recently, numerous microRNAs (miRNAs, ~22 nt) have been shown to be an important mediator and abundantly expressed in a variety of gastric cancers.^28^ Among them, miR-21 is one of the most comprehensive and functional miRNAs and it is highly expressed in many malignant tumours.^29^ Through our experiment, there is no exception that the relative RNA expression of miR-21 was upregulated. Besides, the association between miR-21 and NF-κB has been confirmed by lots of literatures. Upregulated miR-21 in fibroblast-like synoviocytes (FLS) in rheumatoid arthritis (RA) model rats may promote cell proliferation by facilitating NF-κB nuclear translocation, thus affecting the NF-κB pathway.^30^ Herein, the level of miR-21 was decreased with the increasing of NF-κB inhibitor through Chip method, which indicates that miR-21 is regulated by NF-κB. miR-21 also confers Cisplatin resistant in gastric cancer cells, which was coincident with previous studies, for example, exosomal transfer of tumour-associated macrophage-derived miR-21 confers Cisplatin resistance in gastric cancer by regulating PI3K/AKT pathway^31^ and PTEN.^32^

In the present research, luciferase assay was conducted to confirm that caspase-8 is a target of miR-21 was confirmed by luciferase assay, and miR-21 inhibits Cisplatin-induced cell death via negatively regulating caspase-8. Our results were consistent with others’ findings, overexpression of miR-21 suppressed SAOS-2 cells apoptosis via directly targeting caspase-8.^33^ Downregulation of caspase-8 by miR-21 blocks receptor interacting protein-1 cleavage and induces the activation of NF-κB, which regulates these miRNAs.^34^ Additionally, we found LV-METase transfection could suppress expression of miR-21 and NF-κB activity, also increasing expression of caspase-8. All these results suggested that NF-κB/miR-21 pathway was involved in the process of LV-METase enhancing Cisplatin resistant.

In summary, the present study suggested that LV-METase sensitised resistant gastric cancer cells to TRAIL-mediated tumouricidal effect both in vitro and in vivo. upregulation of death receptors, downregulation of anti-apoptotic proteins, and inactivation of NF-κB were involved in the synergistic interaction. However, further studies are being directed toward enhancement of the anticancer effectiveness of LV-METase.

## Electronic supplementary material

Supplementary table

Supplementary Figure 1

Supplementary Figure 2

Supplementary Figure 3
