# Supplementary material for: The mechanism study of lentiviral vector carrying methioninase enhances the sensitivity of drug-resistant gastric cancer cells to Cisplatin
Source: Br J Cancer. 2018 Mar 26;118(9):1189–99. doi: 10.1038/s41416-018-0043-8 (PMC5943323; doi:10.1038/s41416-018-0043-8)
Supplement: Supplementary file 1 — Supplementary table [file 41416_2018_43_MOESM1_ESM.docx]

**WB**

| **Figure 2** | P-gp | | TRAIL | | p-p65 | | DR5 | | c-caspase 8 | | c-caspase 3 | |
| --- | --- | --- | --- | --- | --- | --- | --- | --- | --- | --- | --- | --- |
|  | S | B | S | B | S | B | S | B | S | B | S | B |
| control | 1 | 1 | 1 | 1 | 1 | 1 | 1 | 1 | 1 | 1 | 1 | 1 |
| LV-NC | 0.94 | 1.04 | 1.13 | 0.86 | 0.89 | 1.11 | 1.07 | 1.21 | 0.91 | 1.28 | 0.85 | 0.91 |
| LV-METase | 0.21 | 0.27 | 3.14 | 2.51 | 0.17 | 0.12 | 2.78 | 3.44 | 5.63 | 4.58 | 2.96 | 3.38 |

| **Figure 7** | caspase 8 | | c-caspase 8 | | c-caspase 3 | | P-gp | |
| --- | --- | --- | --- | --- | --- | --- | --- | --- |
|  | S | B | S | B | S | B | S | B |
| control | 1 | 1 | 1 | 1 | 1 | 1 | 1 | 1 |
| miRNC | 1.08 | 1.05 | 0.89 | 0.99 | 0.96 | 1.12 | 1.02 | 1.07 |
| miR-21-5p inhibitor | 2.37 | 1.94 | 3.51 | 3.17 | 4.86 | 4.31 | 0.23 | 0.29 |
| miR-21-5p mimic | 0.39 | 0.13 | N/A | N/A | N/A | N/A | 2.45 | 2.77 |

| **Figure 8** | TRAIL | | p-p65 | | DR5 | | c-caspase 3 | | P-gp | |
| --- | --- | --- | --- | --- | --- | --- | --- | --- | --- | --- |
|  | S | B | S | B | S | B | S | B | S | B |
| control | 1 | 1 | 1 | 1 | 1 | 1 | 1 | 1 | 1 | 1 |
| METase | 3.54 | 4.17 | 0.11 | 0.12 | 6.91 | 5.76 | 5.31 | 7.37 | 0.08 | 0.22 |

Note： S stands for SGC7901/DDP, and B stands for BGC823/DDP

**Primary antibody**

| **Antigen** | **Species** | **Dillution** | **Vendor** | **Catalog** | **MW(Da)** |
| --- | --- | --- | --- | --- | --- |
| P-gp | Rabbit | 1:1000 | Abcam | 168337 | 180 |
| TRAIL | Rabbit | 1:1000 | Abcam | 42121 | 34 |
| DR5 | Rabbit | 1:1000 | Abcam | 47179 | 58 |
| p-p65 | Rabbit | 1:1000 | Abcam | 76302 | 65 |
| Caspase 8 | Rabbit | 1:3000 | Abcam | 32125 | 55 |
| c-caspase 8 | Rabbit | 1:2000 | Abcam | 25901 | 18 |
| c-caspase 3 | Rabbit | 1:200 | Abcam | 2302 | 17 |
| β-actin | Rabbit | 1:200 | Abcam | 16039 | 42 |

**Secondary antibody**

| **Antigen** | **Reporter** | **Dillution** | **Vendor** | **Catalog** |
| --- | --- | --- | --- | --- |
| Goat anti Rabbit | HRP | 1:3000 | Abcam | 205718 |
