# Supplementary figures and images for: The mechanism study of lentiviral vector carrying methioninase enhances the sensitivity of drug-resistant gastric cancer cells to Cisplatin
Source: Br J Cancer. 2018 Mar 26;118(9):1189–99. doi: 10.1038/s41416-018-0043-8 (PMC5943323; doi:10.1038/s41416-018-0043-8)

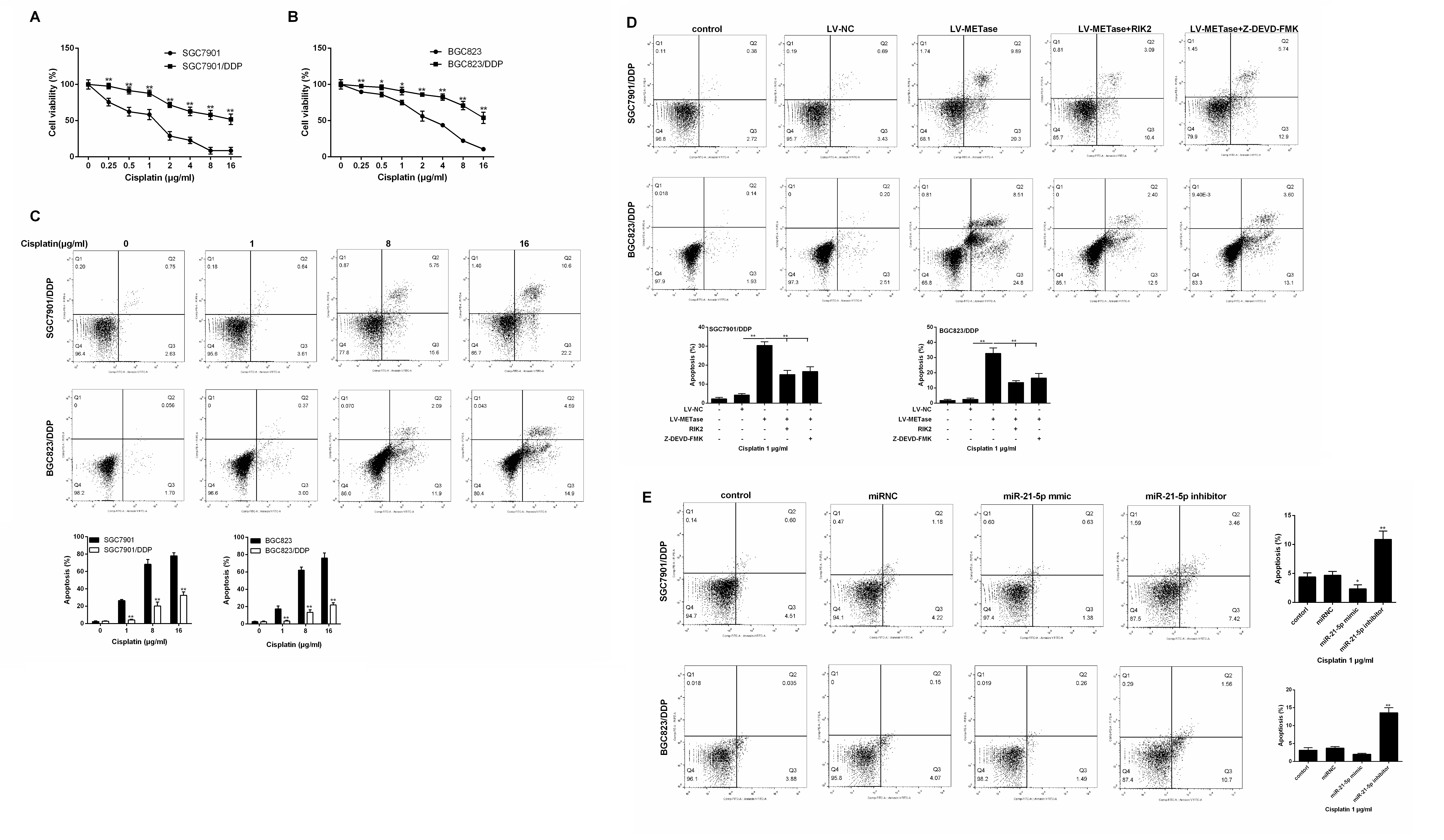

Supplement: Supplementary file 2 — Supplementary Figure 1 [file 41416_2018_43_MOESM2_ESM.tif]

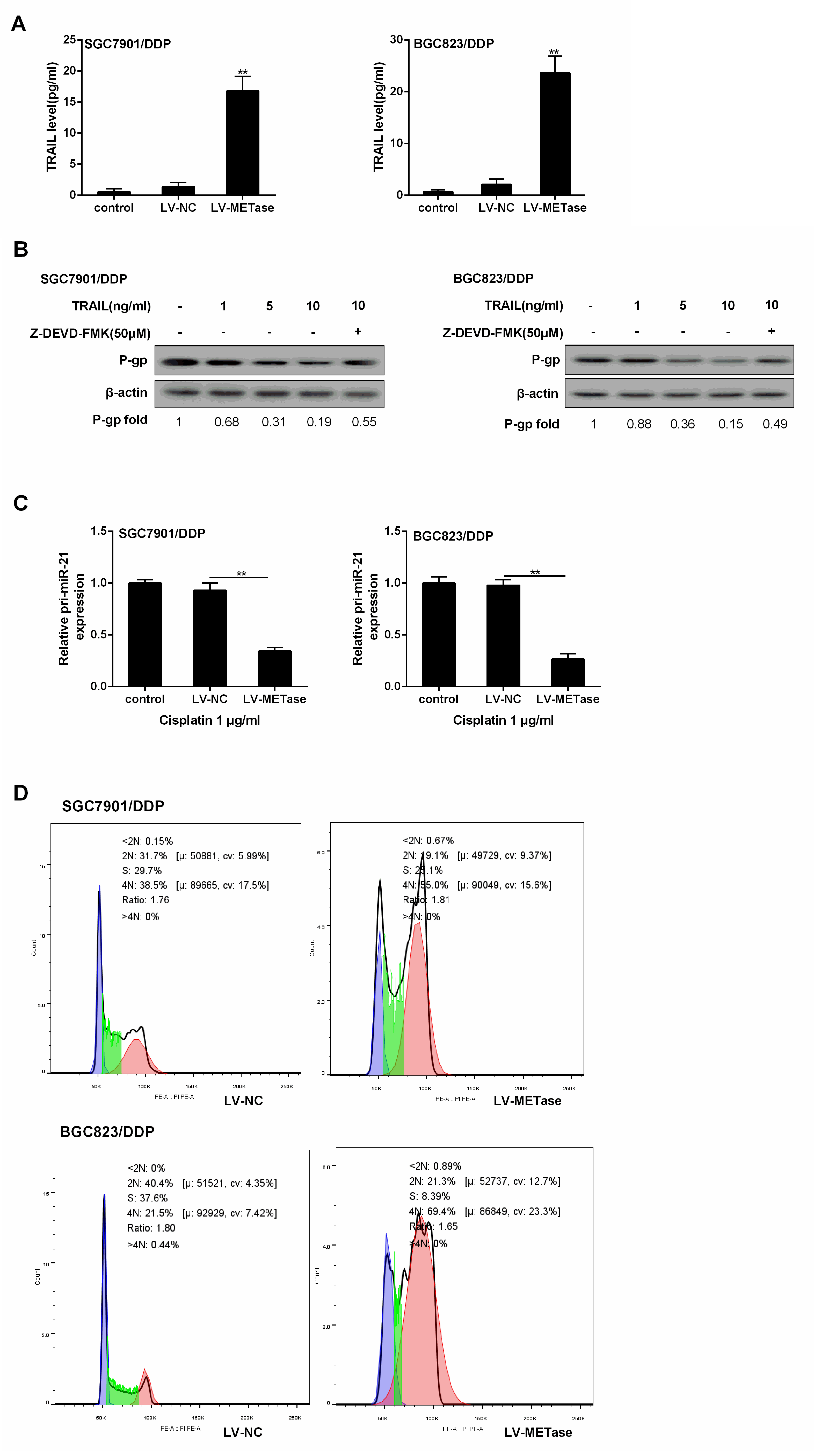

Supplement: Supplementary file 3 — Supplementary Figure 2 [file 41416_2018_43_MOESM3_ESM.tif]
